# Polyphenism – A Window Into Gene-Environment Interactions and Phenotypic Plasticity

**DOI:** 10.3389/fgene.2019.00132

**Published:** 2019-02-26

**Authors:** Chih-Hsiang Yang, John Andrew Pospisilik

**Affiliations:** ^1^ Max Planck Institute of Immunobiology and Epigenetics, Freiburg, Germany; ^2^ Van Andel Research Institute, Grand Rapids, MI, United States

**Keywords:** polyphenism, environment, epigenome, metabolism, obesity

## Abstract

Phenotypic plasticity describes the capacity of a single genotype to exhibit a variety of phenotypes as well as the mechanisms that translate environmental variation into reproducible phenotypic modifications. *Polyphenism* describes the unique sub-type of phenotypic plasticity where the outputs are not continuous, but rather discrete and multi-stable, resulting in several distinct phenotypes on the same genetic background. Epigenetic regulation underpins the stable phenotypic divergences that exemplify polyphenism and their evolutionary origin. Here, we briefly summarize the apparent ubiquity and diversity of polyphenisms across the animal kingdom. We briefly review the best characterized models across taxa and highlight the consistent themes both in their epidemiology and what little we know about molecular mechanisms. Finally, we highlight work that supports the possibility that humans may have a subtle polyphenism at the level of metabolism.

## Introduction

### Phenotypic Plasticity and Polyphenism

Phenotypic output is defined by DNA-sequence (genetics), chromatin regulation (epigenetics and cellular memory) and environmental variables (e.g., nutritional sufficiency), and their interactions (Panzeri and Pospisilik, 2018). Phenotypic plasticity describes the capacity of a single genotype to exhibit a range of phenotypic outputs and the responsiveness of the underlying developmental process to the environment (nutrition, temperature, and population density).

Phenotypic plasticity can be observed across the animal and plant kingdoms. It provides species the possibility to facilitate adaptive changes and increase phenotypic diversity and thus better withstand changes in environment. Phenotypic plasticity is believed to promote the evolution of novelty (Fusco and Minelli, 2010; Sieriebriennikov and Sommer, 2018). Some of the most visually striking examples of phenotypic plasticity are multi-stable in nature including seasonal polyphenism in butterflies (Brakefield and Frankino, 2007); caste polyphenism in eusocial insects (Miura, 2005); defense polyphenisms in cladocerans (*Daphnia cucullata*) (Laforsch and Tollrian, 2004a,b; Miyakawa et al., 2013); temperature and socially regulated sex determination in reptiles (Janzen and Phillips, 2006) and fish (Liu et al., 2017); and environmentally triggered phenotypic plasticity in plants (Gratani, 2014).

Polyphenism is a special case of phenotypic plasticity where development exhibits reproducible bifurcations revealing multiple distinct outcomes (e.g., worker vs. queen bee). Environmental stimuli are the major external triggers of polyphenisms. Multiple physiological pathways, including epigenetic modifications during development, are believed to mediate and maintain phenotypic divergences that exemplify polyphenism. While polyphenisms are by definition discrete, their relative distributions within populations can be either highly stable (e.g., one queen, many workers of the eusocial insect colonies) or highly variable to the extent that entire populations switch or oscillate from 100% of one morph to 100% of the other (e.g., seasonal coloration morphs of the butterfly) (Mayr, 1963). Because essentially all multicellular organisms undergo a developmental process with distinct stages, we do not include developmental stages in our definition of polyphenism as some others have. Similarly, for the purposes of this review, we define epigenetic as chromatin-based mechanisms that can stably transmit transcriptional alterations or “memory” through mitosis (Deans and Maggert, 2015; Greally, 2018). While DNA and histone modifications are most typically discussed in these contexts (Kouzarides, 2007), we will encourage a very broad interpretation of the term here. Given how little we understand polyphenic mechanisms, it is important to include in our thinking modifications of DNA, RNA, histones, and the myriad proteins and metabolites that interact with transcriptional machinery, as well as the signaling circuitry that reinforces transcriptional output from one cell division to the next once an original stimulus has past. In addition, transgenerational effects whether truly epigenetic or whether behaviorally or parentally transmitted should also be kept in mind. In the end, it remains unclear to what extent such switches are continuously reinforced after the completion of development. At least in some instances, polyphenisms are not entirely stable (gamergates, see Section 1).

Below, we highlight the key concepts of what we do understand of the epigenetic and genetic processes driving morph divergences, especially in animal kingdom.

## Polyphenism in Higher Animals

### Insects

#### Caste Polyphenism

Eusocial insects are the epitome of polyphenism; the same genome gives rise to completely different morphologies and behaviors in a population. In this review, we will cover caste polyphenism in three insect species: the Florida carpenter ant (*Camponotus floridanus*), Indian jumping ant (*Harpegnathos saltator*), and the honeybee (*Apis mellifera*).

##### Florida Carpenter Ant (*Camponotus floridanus*)

**Figure 1:**
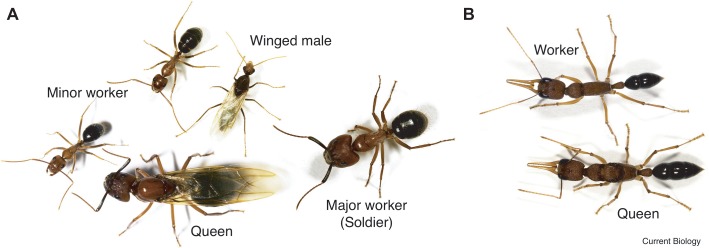
Caste polyphenism in ants. **(A)** *Camponotus floridanus*, a winged (virgin) queen (bottom, center); a (winged) male (top, center), a major worker (soldier) on the right, and two minor workers (bottom left and top left). **(B)** *Harpegnathos saltator*, a worker (top) and queen (bottom). (Reproduced with permission from Chittka et al., 2012).

The castes of Florida carpenter ants are clearly defined in behavior and morphology. Remarkably, each female embryo can potentially become either a minor worker, a major worker, or a queen (Figure 1A; Chittka et al., 2012). Once developed, their fates are fixed. From a behavioral point of view, minor workers forage and scout more than major workers, and the queen is responsible for reproduction. Environmental triggers, such as chemicals, larval nutrition, pheromones, and temperature, all affect the embryo’s caste fate. However, detailed molecular signaling pathways still need to be elucidated. Through genome-wide DNA methylome studies, we now know that the methylation profiles of minor and major workers are more similar to each other than to the queens (Bonasio et al., 2012). Furthermore, DNA methylation occurs in the exons of transcribed genes, which correlates with positive gene expression. Other studies point out that minor and major workers exhibited unique histone acetylation patterns across the genome suggesting that signaling control of histone acetyltransferases or deacetylases (HATs/HDACs) is likely important (Simola et al., 2016). Interestingly, central nervous system (CNS) injection with the class I or class II histone deacetylase inhibitors (HDACi), valproic acid (VPA) or trichostatin A (TSA), both conferred major workers with minor worker-like foraging and scouting activities. Remarkably, histone acetyltransferase p300/CREB-binding protein inhibitor (C646 or EML425) co-injection was found to suppress HDACi-induced foraging and scouting in the major workers.

##### Gamergate in Indian Jumping Ant (*Harpegnathos saltator*)

In the castes of the Indian jumping ant, workers can turn into functional reproductive queens (gamergates) if the original queen dies (Figure 1B; Peeters et al., 2000; Chittka et al., 2012). Data show that the DNA methylation profiles of gamergates are more similar to the workers than to the queen. However, the transcriptomes of gamergates are more similar to queens than to workers (Bonasio et al., 2012). Conceptually, gamergates highlight three important ideas (1) that high-residual plasticity can exist after the developmental switches; (2) some polyphenisms require continuous reinforcement; and (3) polyphenisms can include inter-individual or population-dependent regulation.

##### Honeybee (*Apis mellifera*)

Honeybee castes are the likely the best understood polyphenism. They represent one of many examples where larval or developmental nutrition regulates morph outcome of an individual – either as a worker or as a queen. Larvae fed with relatively low amounts of royal jelly develop into workers, while larvae fed with high amounts of royal jelly develop into queens (Shuel and Dixon, 1960). Silencing the expression of DNA methyltransferase 3 (*Dnmt3*) in newly hatched larvae and in embryos strongly biases an individual’s outcome toward the queen fate (a royal jelly high-mimicking effect). Such queens have fully developed ovaries compared to control-treated individuals (workers) (Kucharski, 2008). These studies highlight the dramatic potential that developmental nutrition can have on developmental fates.

#### Dispersal Polyphenisms

Dispersal polyphenisms are a sub-classification characterized by morph phenotypes that markedly influence the reproductive dispersal of an organism and thus the population (new cohort). A common example is winged versus wingless insect morphs that develop in heterogeneous environments (such as seasonal changes). This alternative dispersal ability is likely advantageous for adapting new habitats and for maximizing focal populations while ensuring population spread and outbreeding under a wide range of environmental possibilities.

##### Wing Polyphenism in Aphids (*Acyrthosiphon pisum*)

**Figure 2:**
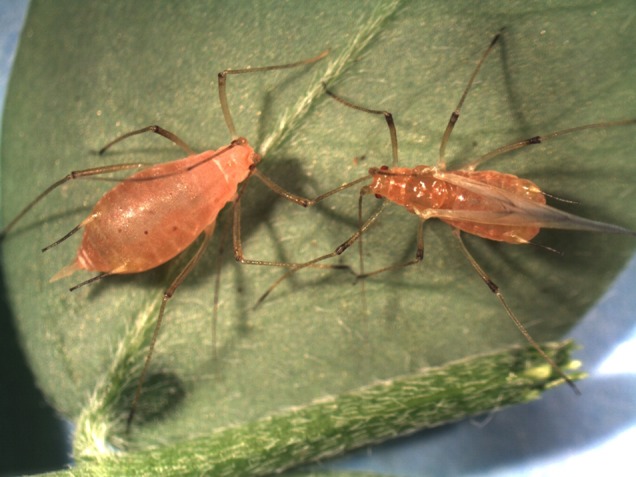
Wing polyphenism in aphids. Winged (right) and wingless (left) forms of female aphids, *Acyrthosiphon pisum* (Reproduced with permission from Simpson et al., 2011).

Aphids are a diverse group of small insects with complex life cycles. Many of them undergo cyclic parthenogenesis and sexual reproduction. Sexual reproduction only occurs at the end of summer when temperatures fall or when daytime starts to shorten. Female aphids have an environmentally determined wing polyphenism (winged vs. wingless) during the parthenogenesis process (Figure 2; Dixon, 1998; Simpson et al., 2011). There are numerous known environmental triggers: population density, host plant quality, temperature, and photoperiod (Müller et al., 2001; Braendle et al., 2006; Brisson, 2010). However, the exact molecular pathways by which these signals are detected and by which they elicit a phenotypic switch remain unresolved. Homologous genes to the most well-known canonical epigenetic regulators do exist in the aphid. Further, epigenome organization includes the regulation of fundamental processes such as DNA methylation at the genome-wide level (Walsh et al., 2010). We do not yet know to what extent epigenome regulation in the aphid correlates or mechanistically underpins the various morphs (Zhang et al., 2019).

### Nematodes

#### Mouth-Form Polyphenism

**Figure 3:**
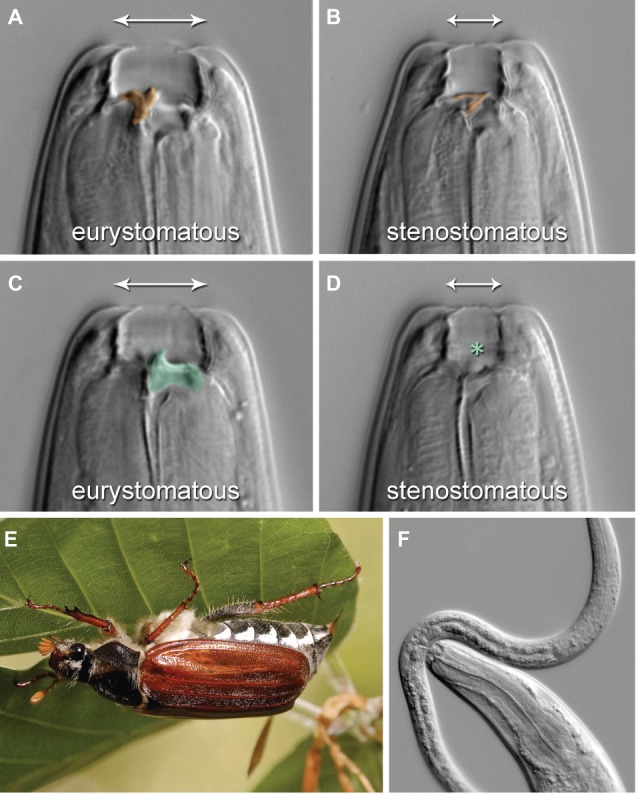
Mouth-form polyphenism in *P. pacificus* Nematodes. **(A,C)** are a single Eu individual in two focal planes, whereas **(B,D)** are a single St individual in the corresponding planes. **(A,B)** are sagittal, **(C,D)** right sublateral planes. The Eu form bears a claw-like dorsal tooth [**(A)**, false-colored orange] and an opposing, claw-like right subventral tooth [**(C)**, green], whereas the St form has a thin, flint-shaped dorsal tooth [**(B)**, orange] and no subventral tooth [**(D)**, asterisk]. The mouth-form polyphenism also includes mouth breadth (indicated by arrows). **(E)** The mouth-form polyphenism imparts novel feeding abilities that foster a necromenic association with scarab beetles, including the European cockchafers (*Melolontha* spp.), cadavers of which are host to diverse microorganisms used as food. **(F)** The complex structures of the Eu form are associated with the ability to prey on other nematodes, as pictured by an Eu *P. pacificus* individual attacking a *Caenorhabditis elegans* larva. (Reproduced with permission from Ragsdale et al., 2013).

*Pristionchus pacificus* (*P. pacificus*) is one of the most well-studied species of nematodes apart from *Caenorhabditis elegans* (*C. elegans*). They can be cultured on bacteria in the laboratory, but they live in a necromenic association with scarab beetles in the wild (Figure 3E). While the polyphenisms of *P. pacificus* have only recently begun to be studied, it is already one of the best understood systems molecularly because of the ease with which the species can be experimentally and genetically controlled, at least under laboratory conditions.

*P. pacificus* undergoes embryonic development within an eggshell followed by post-embryonic development that consists of four juvenile (J1–J4) stages. The life cycle of *P. pacificus* is approximately 4 days at 20°C, but under stressed environmental conditions, such as food shortage or high-population density, J2 larvae form dauers instead of developing into J3 larvae. In the necromenic association with scarab beetles, J2 larvae rest as dauer instead of entering J3 larvae on the insect and resume development into J3 larvae after the beetle’s death – in the carcass – to feed on the growing microbes.

*P. pacificus* has versatile teeth-like denticles and mouth forms that can be either eurystomatous (Eu) or stenostomatous (St) (Figure 3; Ragsdale et al., 2013). The dorsal tooth of Eu individuals is big and has a claw-like shape, allowing the respective individual to successfully bite, kill, and feed on other nematodes (Figure 3A). The dorsal tooth of St individuals is less physically profound and not claw-like; St individuals are therefore much less successful at predation (Figure 3B). Eu individuals also have a ventral tooth, while St do not (Figures 3C,D). The buccal cavity also differs between the two mouth forms in width and height. The fate determination of the mouth form of an individual animal occurs during larval development and is irreversible. Importantly, whereas both Eu and St animals feed on bacteria, Eu individuals rely on a mixed source of food including other nematodes (Figure 3F). This behavior significantly alters the competitive environment with regard to food availability and survival. Selection for either Eu or St animals in an inbred strain for 10 generations did not change the ratio of the two mouth forms in populations of the resulting sub-strains (Bento et al., 2010). These results suggest that this novel morphological feeding structure in *P. pacificus* is an example of polyphenism, rather than deriving from a genetic polymorphism. The known non-genetic mechanisms modulating the Eu or St polyphenic outcome in *P. pacificus* are as follows:

##### Environmental and Conditional Cues

Starvation and pheromones are two identified triggers that increase Eu individuals in a population (Bento et al., 2010). Conversely, isolating individual larvae from a population (a method to mimic low-population density) triggers the St form. Consistent with these data, conspecific crowding promotes the Eu form. Interestingly, mouth form appears to be regulated by maternal epigenetic inheritance. Male offspring born to Eu mothers are highly probable to be St (80%), while those born to St mothers are all essentially St (Serobyan et al., 2013).

##### Developmental Regulation

The nuclear hormone receptor, DAF-12, binds the steroid hormone Δ7-dafachronic acid (Δ7-DA). DAF-12 and Δ7-DA represent the first two molecules identified that specify mouth-form decision of individuals in response to starvation and pheromones. Loss-of-function of DAF-12 in dauer formation defective (daf-d) mutants, which are weakly responsive to starvation and unresponsive to pheromones, decreases the incidence of Eu formation. In addition, Δ7-DA treatment (DAF-12 ligand) decreases the incidence of Eu individuals as well (Bento et al., 2010).

In a forward genetic approach, from Eu-form-defective mutant (eud-1), the authors identified Eud-1 as a developmental switch regulator. Eud-1 mutants do not produce Eu forms, while transgenic lines overexpressing eud-1 produce only Eu forms. One pheromone known to specifically regulate mouth dimorphism, diascaroside dasc#1, fails to turn eud-1 mutant into Eu forms when compared to the wild-type strain (98% Eu). This result indicates that the EUD-1 switch responds to external pheromones.

In an epistasis study, Δ7-DA had no effect on the eud-1 transgenic line (100% Eu). This indicates that EUD-1 regulates mouth dimorphism downstream of Δ7-DA/DAF-12 (Ragsdale et al., 2013). Still, how exactly the pheromones and hormone signals are coupled to EUD-1 and detailed upstream of EUD-1 needs to be answered.

##### Robustness

Different from phenotypic plasticity, robustness or “canalization” of phenotype describes the resistance of phenotypic development to environmental perturbations. The relation of plasticity and robustness has been contrasted importantly as complementary rather than opposing (Sieriebriennikov and Sommer, 2018). Heat-shock protein 90 (Hsp90) is one of the few proteins known to affect robustness, an activity that appears conserved across plant and animal kingdoms (Queitsch et al., 2002; Salathia and Queitsch, 2007). Molecularly the effects mediated by Hsp90 coordinate environmental input with broad scale transcriptional rewiring as well as regulation of intergenerational programming and unmasking of cryptic sequence-dependent regulation. These roles of Hsp90 are worthy of independent review (Rohner et al., 2013; Heard and Martienssen, 2014) and beyond the scope of this review. Relevant for our discussion here though, Sieriebriennikov et al. have briefly examined the relation of robustness and polyphenism with the mouth-form polyphenism in *P. pacificus* as a model (Sieriebriennikov et al., 2017). Using heat-stress treatment, pharmacological inhibition with radicicol, and knockout of the *Hsp90* homolog *ppa-daf-21*, the authors observed that mouth-form polyphenism morph frequencies are largely independent of heat shock perturbations, though they did observe an abnormal shift and expansion of mouth morphology in some circumstances. By definition, discrete phenotypes will be more easily distinguishable if their individual variances are minimized. This balance between constraining phenotypic variation concordant with enabling a developmental switch is central to enabling polyphenism and is still underexplored.

### Fish and Reptiles

#### Sex Determination

Sex determination can be controlled by multiple genetic systems: inheritance of chromosomal composition upon fertilization; environmental conditions like temperature during embryonic stages; and combinations of both as seen in reptiles and fish (Janzen and Phillips, 2006; Liu et al., 2017). For example, in turtles and lizards, apart from genotypic sex determination (GSD), the idea of temperature-dependent sex determination (TSD) is well known (Roush and Rhen, 2018). In part because of the difficulty associated with genetic manipulation of these organisms to date, little is known about the molecular mechanisms that mediate environmental control of sex determination. For this reason, here, we highlight just one of many intriguing examples where some genetic and epigenetic correlates have been mapped: socially induced sex change in Bluehead wrasse.

#### Social Structure-Induced Sex Change

##### Caribbean Bluehead Wrasse (*Thalassoma bifasciatum*)

Bluehead wrasse is small coral reef fish composed of a dominant terminal-phase male and a group of females, as well as some initial-phase males. The initial sex determination is poorly understood. Under conditions where a dominant male is removed from a natural social group, one of the largest females can perform protogynous sex change into a dominate male followed typical dominant behaviors (aggression and courtship) and changes of external morphology and internal gonadal anatomy (Warner, 1984; Warner and Swearer, 1991). Initial males can also transform into dominant males (Liu et al., 2017). The molecular regulation of gonadal sex change in *T. bifasciatum* has been described, at least in part.

Gonadal sex change in the protogynous process involves ovarian atresia followed by testicular growth, a complete ovary-to-testis transition (Cole, 2011). Before sex change, female fish need high estrogen to maintain ovarian function and follicle survival (Lubzens et al., 2010). Production of the estrogen estradiol (E2) relies on the activities of gonadal aromatase (*cyp19a1a* in fish), an enzyme that converts androgens to estrogens (Frisch, 2004). Downregulation of *cyp19a1a* is observed in transitioning gonads (Zhang et al., 2008). The maintenance of gonadal sexual fate involves two key transcription factors Dmrt1 and Foxl2, which are proposed to have antagonistic effects on cyp19a1a expression in fish (Liu et al., 2007; Wang et al., 2010). During the protogynous sex change, *Foxl2* is downregulated (Liu et al., 2015), while *Dmrt1* is upregulated (Liu et al., 2007; Kobayashi et al., 2014). Taken together, the two transcription factors are important for progression of sex change but not for its initiation.

Evidence of dimorphic DNA methylation patterns in the promoter regions of *cyp19a1a* is well observed in fish (Navarro-Martín et al., 2011; Zhang et al., 2013). *In vitro* studies showed that hyper-methylation of *cyp19a1a* promoter regions is inversely correlated to protogynous gonadal sex change and thus suggested to regulate its initiation. How upstream epigenetic modifications are controlled remains unexplored.

### Mammals – Uncharted Territory

Phenotypic bi-stability or polyphenisms have been reported primarily in plants, fish, insects, worms, and reptiles. That said, there have also been reports in mammals. Because of the paucity of such data though, and the vast potential phenotypic plasticity poses for human medicine, we include them here as a perspective, to help open minds to the possibilities and challenges associated with having alternate potentials locked away in our own genomes.

#### Naked Mole Rats *(Heterocephalus glaber)*

Naked mole rats, *Heterocephalus glaber*, live underground in the wild. The pinkish, almost hairless animals have small eyes and can barely see. They have acute hearing, a well-developed sense of smell, and live in colonies.

#### Caste Polyphenism and Dispersal Polyphenism

Naked mole rats are one of the few known mammals that are eusocial (similar to eusocial insects). Naked mole rats exhibit two examples of discrete phenotypic variation. Like insects, strong phenotypic variation is associated with breeding social structure. Colonies have a single breeding queen that is larger, produces impressive numbers of offspring, and relies on servitude of colony workers for food and hygiene. If the queen of the colony dies, a few females may fight to become the new queen. When the new female begins her reign, she grows even though she is already an adult; a striking adult morphological plasticity that parallels ant gamergate plasticity and *bona fide* phenotypic plasticity of the same genome (Sherman et al., 1991; Cohn, 1992; Margulis et al., 1995). Interestingly, under laboratory conditions, non-breeding individuals in the colony can exhibit one of two potential morphs that appear to represent a classic developmental switch polyphenism: they can appear as non-dispersers and dispersers. The vast majority of animals are non-dispersers – “normal” naked mole rats for lack of a better term – which work all day, digging, foraging, serving the queen, and engaging in colony life. The rare disperser morph, by contrast, acts like potential outbreeders. In a mate-choice experiment, dispersers showed a significant preference for unrelated mole rats, while non-dispersers always responded aggressively to the unrelated mole rat. Dispersers have significantly higher levels of bioactive luteinizing hormone in the plasma than non-dispersers, and dispersers form distinct groups within the colony. They have minimal participation in cooperative maintenance tasks and display a better locomotion and feeding activities compared to non-dispersers. Dispersers have a significantly higher total body fat than non-dispersers of similar body mass and age. Fat reserves act as a nutritional safeguard for dispersers against starvation during dispersal and colony finding. The colonies harboring dispersers were significantly larger than colonies without dispersers. With respect to environmental cues for the non-dispersal/dispersal switch, the size, age, and/or composition of colonies matter (Justin O’Riain, 1996). Thus far, nothing is known about the molecular mechanisms underpinning naked mole rat polyphenisms.

#### Mouse (*Mus musculus*)

##### Body Composition Polyphenism

Our own group has demonstrated polyphenisms in the mouse. Building on ambitious experiments by the Whitelaw group intended to identify novel chromatin regulators in mice, we focused on chromatin regulatory mouse mutants with evidence of altered or enhanced phenotypic variation. One MommeD9 (*Trim28^+/D9^*), a non-sense mutation in the chromatin-interacting protein gene *Trim28* (also known as *Tif1b* or *Kap1*) stood out in which they exhibited exaggerated stochastic phenotypic variation specifically in body mass and adiposity (Whitelaw et al., 2010). Careful examination of these effects revealed a non-random, non-Gaussian hyper-variability in *Trim28^+/D9^* mice (Dalgaard et al., 2016), Rather, mutant mice exhibited obesity in an “on/off” manner in isogenic *Trim28^+/D9^* colonies. The result was a bi-modal body-weight distribution for the population. In addition to a tendency toward an increase in body size, the obese murine morph is characterized by downregulation of an imprinted gene network 1 (IGN1) that appears causal in developmental bifurcation. Specifically, obese morphs exhibit reduced expression of neuronatin (*Nnat*), *Peg3*, *Cdkn1c*, and *Plagl1*. Deletion of either *Nnat* or *Peg3* alone is sufficient to recapitulate the bi-modal adiposity phenotype, thus identifying a still small regulatory network of developmental switch genes in mammals and highlighting the stark potential for discrete epigenetic states in mammalian disease. To our knowledge, this work represents the first genetic proof of mammalian polyphenism, though we find evidence of obese morphs at frequencies of ~1–5% in most colonies examined.

Overall, the well-studied examples of polyphenisms in insects confer “obvious” evolutionary advantages, even where they have yet to be tested. BMI/adiposity polyphenism in the mouse might confer multiple evolutionary advantages: difficulties associated with requiring marginally larger burrows might be offset by resistance to famine/food deprivation, reduced necessity to forage under higher-risk conditions, advantage during mate competition, improved lactation and maternal care, improved resistance to cold, physical injury, and drowning. Equally important phenotypic plasticity and bi-stability in particular have been proposed as precursors for establishing evolutionary novelty and speciation.

#### A Case for Human Polyphenism (*Homo sapiens*)

##### Body Composition Polyphenism?

**Figure 4:**
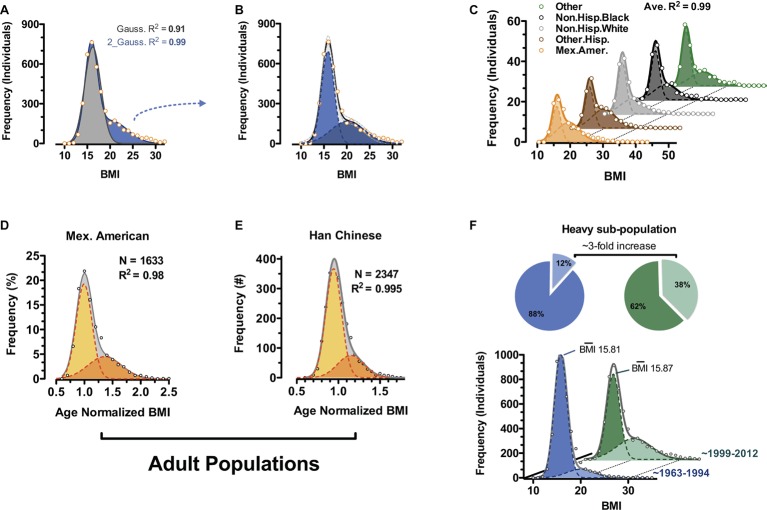
Body mass index bimodality in human. **(A)** BMI distribution of 6- to 11-year-old non-Hispanic white males from the continuous NHANES 1999–2012 survey (CDC and NCHS, 2012). Data are fit to a single Gaussian (gray) and a double Gaussian (blue). **(B)** Individual Gaussian components of the double Gaussian from **(A)**. **(C)** Near-perfect double-Gaussian fit is observed across children of five major ethnicity classes, as well as in adult cohorts from **(D)** continuous NHANES 1999–2012 (CDC and NCHS, 2012) and **(E)** Han Chinese populations. **(D,E)** Shown are age-normalized BMI distributions for females aged 25–50. **(F)** Comparison of similar fitting of childhood data from continuous NHANES 1999–2012 (CDC and NCHS, 2012) and prior NHANES/NHES surveys (1963–1994) (CDC and NCHS, 1994) shows a marked shift in recent decades where the heavy sub-population triples in size (pie charts). (Reproduced with permission from Dalgaard et al., 2016).

Unbiased examination of publically available epidemiology datasets reveals bimodality at least in human body weight distributions and specifically body mass index (BMI) (Figure 4; Dalgaard et al., 2016). Bimodality can be observed in children of all recorded ethnic classes of the National Health and Nutrition Examination Survey (NHANES) 1968–2012 survey (Figures 4A–C; CDC and NCHS, 2012) and in select adult cohorts (Mexican American and Han Chinese) (Figures 4D,E). Rather than what has often been reported as a “positively skewed” Gaussian distribution for BMI, our analyses using two independent and potentially overlapping Gaussian sub-populations fit most ethnically homogenous human BMI distributions to a greater than 98%. Comparing BMI distributions from NHANES data gathered between 1963 and 1994 (CDC and NIHS, 1994) with the more recent 1999–2012 (continuous NHANES) data (CDC and NCHS, 2012), the frequency of individuals within the heavy sub-population of BMI triples while that of the lighter sub-population decreases (Figure 4F; Dalgaard et al., 2016; 1999–2012 NHANES vs. 1963–1994 NHANES). Remarkably, it is virtually exclusively a change in frequencies, as the magnitude of BMI for each of the sub-populations remains essentially fixed over the same timescale. These data suggest increased incidence of a distinct category of “triggered” individuals consistent with the rodent obese and dispersal morphs described above. They argue against the general statement that the whole population is becoming much more obese. Importantly, the data are in total agreement with the notion of polyphenism.

#### Evidence of *TRIM28*/IGN1-Associated Obesity in Select Human Cohorts

Beyond epidemiology, our studies have also taken us to investigations of gene expression in a cohort of 22 lean and 18 obese children, aged ~2–10 years of age, a time of relatively tight environmental control in human lifespan (Kathrin Landgraf et al., 2015; Dalgaard et al., 2016). *TRIM28* is significantly reduced in adipose tissue of obese children. To mimic the analysis of *Trim28^+/D9^* haploinsufficiency in mice, all individuals were grouped into high or low *TRIM28* expressors based on adipose tissue mRNA levels and sub-grouped into obese or lean groups. Interestingly, in contrast to high *TRIM28* expressors, obese children with low Trim28 also exhibited lower IGN1 gene expression when compared to equally *TRIM*28-low groups of lean subjects. These results recapitulated IGN1 downregulation in *Trim28^+/D9^* haploinsufficient mice and are consistent with a conserved role for a *TRIM28*-IGN1 axis in human adiposity regulation. Perhaps most interesting, principal component analysis of transcriptomes from all the same individuals indicated that the components of the greatest variation across the cohort were not obese vs. lean, or male vs. female individuals, but rather *TRIM28*-low vs. *TRIM*28-high individuals indicating substantial differences exist between these two as of yet ill-defined categories of humans.

While these childhood cohort studies intrigue, they remain poorly genetically controlled. Adipose tissue microarray data from 13 discordant monozygotic (MZ) twin pairs, each comprising one obese and one normal co-twin (Pietiläinen et al., 2008), indicate that similar patterns of dysregulation may drive “purely” epigenetic dimorphisms in man. *TRIM28* and IGN1 expressions are specifically decreased in the obese relative to lean isogenic co-twins. This indicates that *TRIM28*-IGN1 expression at the least correlates with epigenetic human obesity. If sufficiently powered they would be consistent with *TRIM28*-IGN1 insufficiency as a developmental gene switch.

## Conclusions and Perspectives

Polyphenism is conserved in species from insects to nematodes, and evidence is mounting that it extends into many mammalian species.

The naked mole rat eusocial phenotypic plasticity, whose phenotypes are still malleable after adulthood, is reminiscent of seasonal phenotypic plasticities in coat colors of arctic animals and in antler growth. It is similarly reminiscent of physical attributes associated with alphas in pack animals, a social and physiological divergence that is widespread across the mammalian kingdom. The blurriness between the concepts of polyphenism and hierarchy-associated phenotype reflects our limited understanding of the interplay between environment (social or physical) and the molecular regulation of developmental switches. It will be intriguing to see the overlap and contrasts in molecular mechanisms that beget adult seasonal/hierarchical phenotypic plasticities. The seemingly more stable developmental switches likely reflect analogous molecular principles at earlier developmental time points and therefore with much farther reaching and stable developmental consequences. Will we find strong concordance in molecular principles between socially reinforced phenotypic differences (alphas and wrasse), robust nutritionally conditional systems (royal jelly of the queen bee), and classical developmental switch polyphenisms? Considering what we know of the enormous diversity that nature presents, there is no reason that these concepts should be mutually exclusive.

The environmental and mechanistic underpinnings that contribute toward polyphenism in mammals are only beginning to be elucidated. Although we have anecdotal evidence that *Trim28^+/D9^* mice show a reduction in the frequency of obese morphs with increased housing density and reduced environmental temperatures, these have yet to be formally proven and the mechanistic nature of the triggers needs to be investigated. When and how are the developmental switches regulated? *Trim28* loss has been linked to the dysregulation of imprinted genes during development (Messerschmidt et al., 2012). IGN1 genes have been implicated in placentation (Sekita et al., 2008), development, and growth. Mutating both insulin and insulin-like growth factor 1 (IGF1) receptors leads to reduced expression of a subset of IGN1 imprinted genes (Boucher et al., 2014), suggesting insulin and IGF1 signaling as potential regulatory candidates for generating bi-stability during embryonic development. Furthermore, *NNAT*, as one gene of the IGN1 network, has been correlated with human obesity (Vrang et al., 2010; Gburcik et al., 2013) and very recently with regulation of hormone maturation in endocrine cells (Millership et al., 2018). Two single-nucleotide polymorphisms (SNPs) within the *NNAT* locus are associated with susceptibility to severe forms of obesity in human children and adults (Vrang et al., 2010). Further, *NNAT* gene expression is reduced in subcutaneous adipose tissue from obese humans (Gburcik et al., 2013). Further, while a *TRIM28*/IGN1 axis has been defined as the core developmental switch, the upstream signaling linking this axis to the external environment, and the epigenetic regulation that builds bi-stability into the system are the key future questions. Trim28 links DNA methylation, repressive histone modifications, and transcriptional repression. Our own observations indicate that *Dnmt3a^−/+^* mice show not only excessive phenotypic variation (Whitelaw et al., 2010) but also a pattern consistent with a polyphenism of “obese” and “lean” morphs (Yang and Pospisilik et al. unpublished). These data, taken together with the observations of Dnmt-regulated polyphenism in the honeybee, suggest that an evolutionarily conserved system is at play.

A further understanding of the mechanistic principles across species will in the long run solidify our understanding of phenotypic diversity across species, kingdoms, and even across evolution. Hopefully deeper understanding of these magnificent processes can enlighten us equally regarding the non-genetic drivers of human disease.

## Author Contributions

C-HY and JP contributed to the conception and design of the review. C-HY wrote the first draft of the manuscript. All authors revised, read, and approved the submitted version of the manuscript.

### Conflict of Interest Statement

The authors declare that the research was conducted in the absence of any commercial or financial relationships that could be construed as a potential conflict of interest.
